# Sex Differences in the Adverse Electromechanical Remodeling of the Heart After Repeated Witness Stress in Adult Rats: Relationship With a Specific miRNA Signature

**DOI:** 10.1097/PSY.0000000000001406

**Published:** 2025-05-23

**Authors:** Margherita Barbetti, Iolanda Bilotti, Donald Ielpo, Rocchina Vilella, Caterina Frati, Valeria Naponelli, Diego Andolina, Luisa Lo Iacono, Andrea Sgoifo, Monia Savi, Luca Carnevali

**Affiliations:** Department of Chemistry, Life Sciences and Environmental Sustainability, University of Parma (Barbetti, Bilotti, Vilella, Frati, Sgoifo, Savi, Carnevali), Parma; Department of Psychology, Sapienza University (Ielpo, Andolina); IRCCS Fondazione Santa Lucia (Ielpo, Andolina, Lo Iacono), Rome; Department of Medicine and Surgery, University of Parma, Parma (Naponelli); Department of Translational Research and of New Surgical and Medical Technologies, University of Pisa (Lo Iacono), Pisa, Italy

**Keywords:** social stress, sex differences, microRNAs, witness stress, heart, **ANOVA** = analysis of variance, **APB** = atrial premature beats, **AV** = atrioventricular, **BP** = blood pressure, **CTR** = control, **CVD** = cardiovascular disease, **Cx-43** = connexin-43, **ECG** = electrocardiogram, **IVCT** = isovolumic contraction time, **LVEDP** = left ventricular end diastolic pressure, **LVRT** = left ventricular relaxation time, **LVSP** = left ventricular systolic pressure, **miRNAs** = microRNAs, **MPI** = myocardial performance index, **WS** = witness stress, **SEM** = standard error of the mean, **SIRT-1** = sirtuin-1

## Abstract

**Objective:**

Sex disparities in the association between psychosocial stress and cardiovascular risk have been reported, yet the extent to which social stress impacts cardiovascular function in a sex-specific manner remains unclear. The objectives of this study were to investigate sex differences in the electromechanical remodeling of the heart of socially stressed rats, and to explore potential epigenetic mechanisms (cardiac microRNAs).

**Methods:**

Adult wild-type Groningen rats of both sexes vicariously experienced the social defeat bout between 2 males for 9 consecutive days [“witness stress” (WS) paradigm] or were exposed to a control condition (*n*=8/sex/group). After repeated WS exposure, arrhythmic vulnerability was evaluated via beta-adrenergic stimulation with isoproterenol in conscious rats, while cardiac contractile properties were assessed via hemodynamic analyses under anesthesia. Cardiac tissue was collected to measure expression levels of several miRNAs (including miR-22 and miR-34a) and their molecular targets (eg, SIRT1) involved in the regulation of cardiac electromechanical function.

**Results:**

An increased vulnerability to isoproterenol-induced arrhythmias was found in male, but not female, rats with a history of WS. Signs of contractile dysfunction were found in both sexes after WS, but to a greater extent in males. In addition, only male WS rats exhibited a significantly higher cardiac expression of miR-34a and miR-22, which were associated with a reduced expression of their common molecular target (SIRT-1).

**Conclusion:**

These findings suggest an epigenetic mechanism underlying the larger vulnerability to adverse cardiac electromechanical remodeling in socially stressed male rats, informing our understanding of the sex-specific impact of social stress on cardiac function.

## INTRODUCTION

Research suggests the existence of potential sex disparities in the association between psychosocial stress exposure and cardiovascular disease (CVD) risk, yet the importance of sex in this link remains understudied, and the limited clinical findings available so far have been inconsistent.^1^ For instance, challenging the traditional view of CVD as a men’s disease, some studies indicate a larger prevalence of stress-induced cardiomyopathy and a higher risk of developing myocardial ischemia following mental stress in women compared with men.^2–4^ Conversely, other studies indicate that chronic mental stress has a stronger effect on endothelial dysfunction, a major predictor of atherosclerosis, in young healthy men compared with age-matched women,^5^ and that the lifetime risk of stress-related coronary heart disease is higher in men compared with women.^6^

While clinical studies offer a more epidemiological perspective, preclinical research is essential for exploring sex-specific cardiovascular vulnerabilities to psychosocial stress and the underlying pathophysiology. For example, the negative cardiovascular impact of social stress exposure has been well-documented in male rodents, primarily using the social defeat model.^7^ Male rodents repeatedly exposed to aggressive male conspecifics develop adverse remodeling of the electromechanical and structural properties of the heart,^8–13^ which may represent a predisposition to CVD. In contrast, the association between social stress and cardiovascular alterations has been less explored in female rodents. This gap likely reflects 2 key factors: (i) driven by the higher prevalence of stress-related psychopathologies in women,^14^ rodent research in females has predominantly focused on the neurobiological effects of social stress by employing stress paradigms like social isolation^15–17^ or social instability^18,19^; (ii) social defeat is challenging to implement in females,^20,21^ hindering direct comparisons between male and female rodents.

To overcome the latter, rodent research has introduced a “witness stress” or “vicarious social defeat” model,^22,23^ in which the physical agonistic interaction between 2 male rodents serves as a source of stress for another rodent (of either sex, the “witness”) placed safely behind a clear perforated partition. Notably, both male and female rats show potent cardiovascular responses to witness stress,^24,25^ as well as several sex disparities in the behavioral and neuroendocrine changes induced by repeated exposure.^26^ Based on these considerations, the primary objective of the current study was to investigate the extent to which the same social stressor (witness stress) impacts the electrical and mechanical properties of the heart of adult rats of both sexes.

Furthermore, we attempted to elucidate potential epigenetic mediators of these sex disparities, namely cardiac microRNAs (miRNAs). miRNAs are small noncoding RNAs, acting as post-transcriptional regulators of gene expression and playing critical roles in both stress responses^27^ and pathological outcomes, including CVDs.^28^ Specifically, we have recently shown that the negative cardiac effects of social defeat stress in male rats are associated with increased cardiac levels of miR-34a,^12^ a key player in the pathophysiological consequences of stress exposure.^29–32^ On the contrary, no changes in cardiac miR-34a levels were found in female rats exposed to repeated witness stress,^33^ suggesting that miR-34a may represent an epigenetic mediator of sex-specific cardiac vulnerability to social stress. However, direct comparisons between these studies are limited by the use of different social stress paradigms (social defeat in males and witness stress in females). Therefore, the second objective of the current study was to investigate whether cardiac alterations induced by repeated witness stress are associated with changes in the expression of candidate miRNAs in a sex-specific manner. We did not limit our investigation to miR-34a but also explored other miRNAs which have been shown to play a crucial role in cardiac electrical and contractile functions, and several pathological cardiac conditions, namely mir-1, mir-22, mir-25, mir-133, mir-208, and mir-328.^34–38^

## METHODS

### Animals

Three-month-old male and female wild-type Groningen rats (*Rattus norvegicus*) were used for this study. This rat strain, originally derived from the University of Groningen (the Netherlands) and now bred at the University of Parma (Italy), is characterized by a higher cardiovascular stress reactivity compared with other rat strains (eg, Wistar rats).^39^ Relatedly, female and male wild-type Groningen rats show similar heart rate and heart rate variability changes during witness stress exposure.^26^ Rats of both sexes were randomly assigned to witness stress (WS, *n*=8/sex) or control (CTR, *n*= 8/sex) groups and were housed individually. Older (8-mo-old) male wild-type Groningen rats were housed in a separate room with an oviduct-ligated female partner and served as residents in the WS paradigm (see below for details). In the same room, an additional 3-month-old male wild-type Groningen rats were used as intruders in the WS paradigm. These animals were housed individually to avoid stress buffering phenomena that occur when conspecific animals are together, which could have altered the WS experience of experimental animals.^40^ All rats were kept in climate-controlled rooms (22±2 °C), with a 12-hour light cycle (lights on at 7 pm), and had free access to food and water. Experimental procedures were performed in accordance with the European Community Council Directive 2010/63/UE and approved by the Italian legislation on animal experimentation (D.L. 04/04/2014, n. 26, authorization no. 473/2022). We referred to the ARRIVE checklist when writing our report.^41^

### General Experimental Protocol

WS and CTR rats of both sexes were implanted under isoflurane anesthesia (2% in 100% oxygen) (Zoetis, Italy) with radio-telemetric transmitters (TA11CTA-F40, Data Sciences International, St. Paul, MN) for electrocardiogram (ECG) recordings (sampling frequency 1000 Hz), according to a previously described procedure.^42^ After surgery, animals were housed individually and received injections of an anti-inflammatory and analgesic drug (Megluflen, Izo, Italy, 0.2 mL/kg, s.c.) for 2 days. After 2 weeks of recovery from surgery, experimental animals were exposed on consecutive days to 9 episodes of WS or CTR procedure. Twenty-four hours after the last WS/CTR episode, animals underwent a proarrhythmic pharmacological challenge, which was followed 2 days later by hemodynamic assessment. Immediately after, hearts were quickly excised and perfused with a 0.9% NaCl solution to flush the residual blood from the tissue, flash frozen in liquid nitrogen, and stored at −80 °C until molecular analyses. Specific experimental procedures are outlined in the next paragraphs.

### Witness Stress and Control Procedures

In the WS paradigm, originally described in rats by,^22^ experimental rats of both sexes (“witnesses”) vicariously experience the social defeat of a male conspecific (the “intruder”) by an older aggressive male rat (the “resident”) behind a clear partition placed in a safe compartment within the resident cage. In this study, resident rats were screened for aggressiveness towards male unfamiliar intruders, and only those showing an attack latency <120 seconds were selected and used for the WS paradigm. On the day of the experiment, the resident’s female partner was temporarily removed from the cage, which was then divided into two compartments of equal size, with the resident rat confined in one of them. Subsequently, the witness rat was introduced to the opposite side, while the intruder rat was placed in the resident’s compartment, where it was physically attacked and socially defeated. The witness rat was exposed to the social defeat of the intruder through sensory (visual, olfactory, and auditory) cues via the clear perforated partition. After 15 minutes, both the intruder and witness rats were returned to their home cages. This procedure was repeated daily for 9 consecutive days, between 10:00 and 11:00 am, under red light. Each time, WS rats witnessed the social defeat of the same intruder but in the cage of a different resident, following a rotational design. On the same day, CTR rats were placed for 15 minutes behind a perforated Plexiglas partition in a novel empty cage (similar to that employed for the WS procedure) with clean bedding. Importantly, the average number of attacks toward the intruder did not differ when the witness was a male or a female (male witness: 5.2±0.6 and 5.4±0.3 during the first and last episode, respectively; female witness: 5.6±0.9 and 5.5±1.1 during the first and last episode, respectively).

### Arrhythmic Vulnerability

Twenty-four hours after the last WS/CTR episode, conscious rats were injected with the drug isoproterenol (β-adrenoceptor agonist, Sigma, St. Louis, MO) at a dose (0.2 mg/kg, i.p.) known to induce arrhythmias in rats.^43^ ECG signals were recorded during the hour that preceded and the hour that followed drug administration. Arrhythmic events were identified via visual inspection of ECG signals using ChartPro 5.0 software by a trained experimenter, who was blinded to the animal’s sex and group.^44^ Specifically, arrhythmic events were assigned point values based on^45^: (a) 1 point for isolated supraventricular events (atrial premature beats or atrioventricular block) or an isolated premature ventricular complex; (b) 2 points for supraventricular tachycardia or 2 or 3 consecutive premature ventricular complexes (SALVO); (c) 3 points for premature ventricular beats in a trigeminal (trigeminy) or bigeminal (bigeminy) pattern or nonsustained ventricular tachycardia (defined as ≥3 and ≤10 consecutive premature ventricular contractions); (d) 4 points for sustained ventricular tachycardia (defined as >10 consecutive premature ventricular contractions) or asystole (absence of electrical activity during the loss of 2 or more consecutive cardiac cycles as defined by the length of the 2 preceding cardiac cycles). Using these categories, an arrhythmic burden was calculated for each rat, defined as the sum of the scored arrhythmic events during the hour that followed isoproterenol injection. In addition, we separately analyzed the total number of arrhythmias for each category.

### Hemodynamic Assessment

Three days after the last WS/CTR episode, rats underwent hemodynamic assessment under ketamine chloride (Imalgene, Merial, Milan, Italy; 40 mg/kg i.p.) plus medetomidine hydrochloride (Domitor, Pfizer Italia S.r.l., Latina, Italy; 0.15 mg/kg i.p.) anesthesia. A microtip pressure transducer catheter (Millar SPC-320, Millar Instruments, Houston, TX) connected to a recording system (Power Laboratory ML 845/4 channels, 2Biological Instruments, Besozzo, Italy) was inserted into the right carotid artery to collect arterial systolic and diastolic blood pressures. The transducer was then advanced into the left ventricle to measure: left ventricular systolic pressure (LVSP) and end-diastolic pressure (LVEDP), the peak rate of rise (+dP/dt_max_) and decline (−dP/dt_max_) of left ventricular pressure (indexes of myocardial efficiency), isovolumic contraction time (IVCT), ejection time (approximated as the time interval between aortic valve opening and time of −dP/d_tmax_), and left ventricular relaxation time (LVRT, computed from −dP/dt_max_ to 5 mm Hg above LVEDP and taken as index of isovolumic relaxation) using the software package AcqKnowledge 3.9 (Biopac Systems, Goleta, CA). In addition, we calculated the myocardial performance index (MPI) as the ratio between total time spent in isovolumic activity (IVCT+ LVRT) and ejection time.^46^ MPI is considered to reflect global cardiac function and provide prognostic information regarding the risk associated with several pathologic conditions, such as myocardial hypertrophy and infarction.^46^ MPI has an inverse relation with global cardiac function, with higher values indicating poorer global ventricular function.

### RNA Purification and Measurement of miRNA Levels

From frozen hearts, punches of the left ventricles were dissected from the midventricular area using stainless-steel tubes of 1 mm inside diameter. Total RNA was extracted from the tissue punches using the total RNA purification Kit (NorgenBiotek, Thorold, Canada) according to the manufacturer’s protocols. RNA quantity was determined by absorbance at 260 nm using a NanoDrop UV-Vis spectrophotometer. The relative expression of miRNAs was measured through quantitative real-time RT-PCR (qPCR), using the Quant Studio 3 thermal cycler apparatus equipped with the SDS software (Applied Biosystems, Waltham, MA) for data collection. Specifically, miRNAs were reverse transcribed using the TaqMan MicroRNA Reverse Transcription kit (#4366596, Applied Biosystems) and then amplified according to the manufacturer instructions, through the correspondent specific TaqMan MicroRNA assays (#4427975, Applied Biosystems): rno-mir1-5p (ID: 001351); hsa-miR-22-5p (ID: 002301); rno-miR-25-5p (ID: 002080); Hsa-miR34a (ID: 000426); rno-miR-133a-5p (ID: 464986_mat); mmu-miR-208a-5p (ID: 462036_mat); hsa-miR-328-3p (ID: 000543); snoRNA (ID: 001718). Ct values were normalized to the averaged measures of sno135. All data were run in triplicate and were expressed as fold changes versus the male control group, according to the 2^−ΔΔC(t)^ method.^47^

### Protein Extraction and Western Blot Analysis

To further investigate the molecular basis of sex differences in the cardiac outcomes of WS, we determined cardiac levels of connexin-43 (Cx43), which plays a key role in the propagation of electrical activity through cardiomyocytes,^48^ and sirtuin-1 (SIRT-1), which has been implicated in the pathogenesis of cardiovascular disease^49^ and represents a common target for both miR-34a^50^ and miR-22.^51^ For protein extraction, frozen pellets of LV cardiomyocytes were homogenized in 400 μL of ice-cold modified RIPA buffer (50 mM Tris HCl pH 7.4, 100 mM NaCl, 1% Triton X-100), supplemented with 1 µL of protease and phosphatase inhibitor cocktails (Merck KGaA, Darmstadt, Germany) every 100 µL of RIPA buffer. Protein concentration was estimated by the DC Protein assay kit (Bio-Rad, Hercules, CA) using bovine serum albumin (Merck KGaA) as a standard. For each animal (*n*=4/5 per group), the equivalent of 50 μg of total protein lysate was loaded and resolved by 15% sodium dodecyl sulfate-polyacrylamide gel electrophoresis. For Western blot analysis, proteins were electrophoretically transferred onto a polyvinylidene fluoride membrane (EMD Millipore, Merck KGaA). Transfer occurred overnight at 4 °C and its efficiency was routinely monitored by 0.1% Ponceau S staining (Merck KGaA). After blocking in a solution of 5% milk for 2 hours at room temperature, blotted membranes were probed overnight at 4 °C with the primary antibodies diluted in TBS-T (50 mM Tris-HCl pH 7.5, 150 mM NaCl, 0.1% Tween 20) containing 5% (w/v) nonfat dry milk. The following antibodies were used: rabbit polyclonal anti-Connexin 43 (Abcam, Cambridge, UK, code ab11370), dilution 1:8000 and rabbit monoclonal anti-SIRT-1 (Abcam, code ab189494), dilution 1:5000. As a loading control, β-actin (GeneTex, Irvine, CA, code GXGTX109639) was used. Following incubation, the membranes were washed 3 times with TBS-T and incubated with horseradish peroxidase-conjugated anti-rabbit (dilution 1:5000) secondary antibodies (Merck KGaA) for 1 hour at room temperature. Immunoreactive bands were detected using the BM Chemiluminescence Western Blotting Substrate (Hoffmann-La Roche, Basel, Switzerland). The optical densities (data were acquired as arbitrary area values) were normalized to those of β-actin and calculated as target protein expression/β-actin expression ratios using ImageJ software version 1.53 (NIH, Bethesda, MD).

### Statistical Analyses

Statistical analyses were performed using the IBM SPSS statistical package (International Business Machines Corporation, Armonk, NY, version 29.0.1.0). Normal distribution of variables was checked by means of the Kolmogorov–Smirnov test. Mean heart rate values during isoproterenol challenge were analyzed using a repeated measures ANOVA test, with “time” (2 levels: before and after injection) as the within-subject factor and “group” (2 levels: WS or CTR) and “sex” (2 levels: males or females) as the between-subject factors. Arrhythmic, hemodynamic, and miRNA data were analyzed using 2-way ANOVA with “group” (2 levels: WS or CTR) and “sex” (2 levels: males or females) as the between-subject factors. Follow-up analyses were conducted using the Student *t* tests with a Bonferroni correction for multiple comparisons. Independent *t* tests were used to compare SIRT-1 and Cx-43 cardiac levels between WS and CTR rats for both sexes. The statistical significance was set at *P*<0.05. Data are reported as means±standard error of the mean (SEM).

## RESULTS

### Sex-specific Effects of WS on Arrhythmic Vulnerability After Isoproterenol Challenge

**FIGURE 1:**
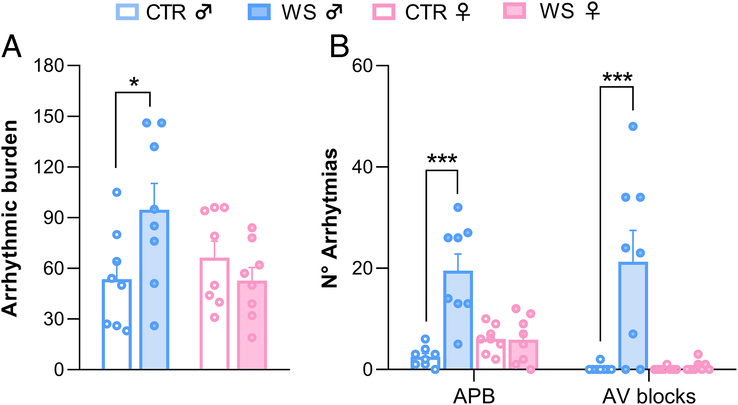
Arrhythmic vulnerability after WS stress. (A) Arrhythmic burden and (B) total number of atrial premature beats (ABP) and atrioventricular (AV) blocks during the hour that followed isoproterenol injection in rats of both sexes (♂: males; ♀: females) exposed to repeated episodes of witness stress (WS) or control (CTR) procedure (*n*=8 per group). Data are reported as means±SEM. Only within-sex effects are reported (*p<.05; ***p<.001). Color image is available only in online version.

Vulnerability to cardiac arrhythmias following isoproterenol challenge in WS and CTR rats of both sexes is depicted in Figure 1. Two-way ANOVA yielded a significant “group×sex” interaction (*F*_(1,28)_ = 5.49, *p*=.026) for the total arrhythmic burden, with WS male rats showing a significantly higher burden compared with CTR males (*p*=.014) and WS females (*p*=.012). There was no difference in the arrhythmic burden between WS and CTR females. As shown in Figure 1B, in males the difference was mainly due to an effect of WS on the incidence of atrial premature beats (group: *F*_(1,28)_=18.79, *p*<.001; sex: *F*_(1,28)_=6.76, *p*=.015; group×sex: *F*_(1,28)_=19.35, *p*<.001) and atrioventricular blocks (group: *F*_(1,28)_=11.93, *p*=.002; sex: *F*_(1,28)_=11.11, *p*=.002; group×sex: *F*_(1,28)_=10.85, *p*=.003). Specifically, WS male rats displayed a significantly higher number of atrial premature beats and atrioventricular blocks compared with their respective CTRs and WS females (*p*<.050 for both). No significant sex and/or group differences were found for the other arrhythmic categories (Table S1, Supplemental Digital Content, http://links.lww.com/PSYMED/B99). In addition, the mean HR recorded for the hour that preceded and the one that followed isoproterenol injection is reported in Table S2, Supplemental Digital Content, http://links.lww.com/PSYMED/B99. Two-way repeated measures ANOVA indicated a significant effect of time, with all animals showing significantly higher mean HR after isoproterenol injection compared with baseline (*p*<.001), independently from sex or group.

### Sex-specific Effects of WS on Hemodynamic Parameters

**FIGURE 2:**
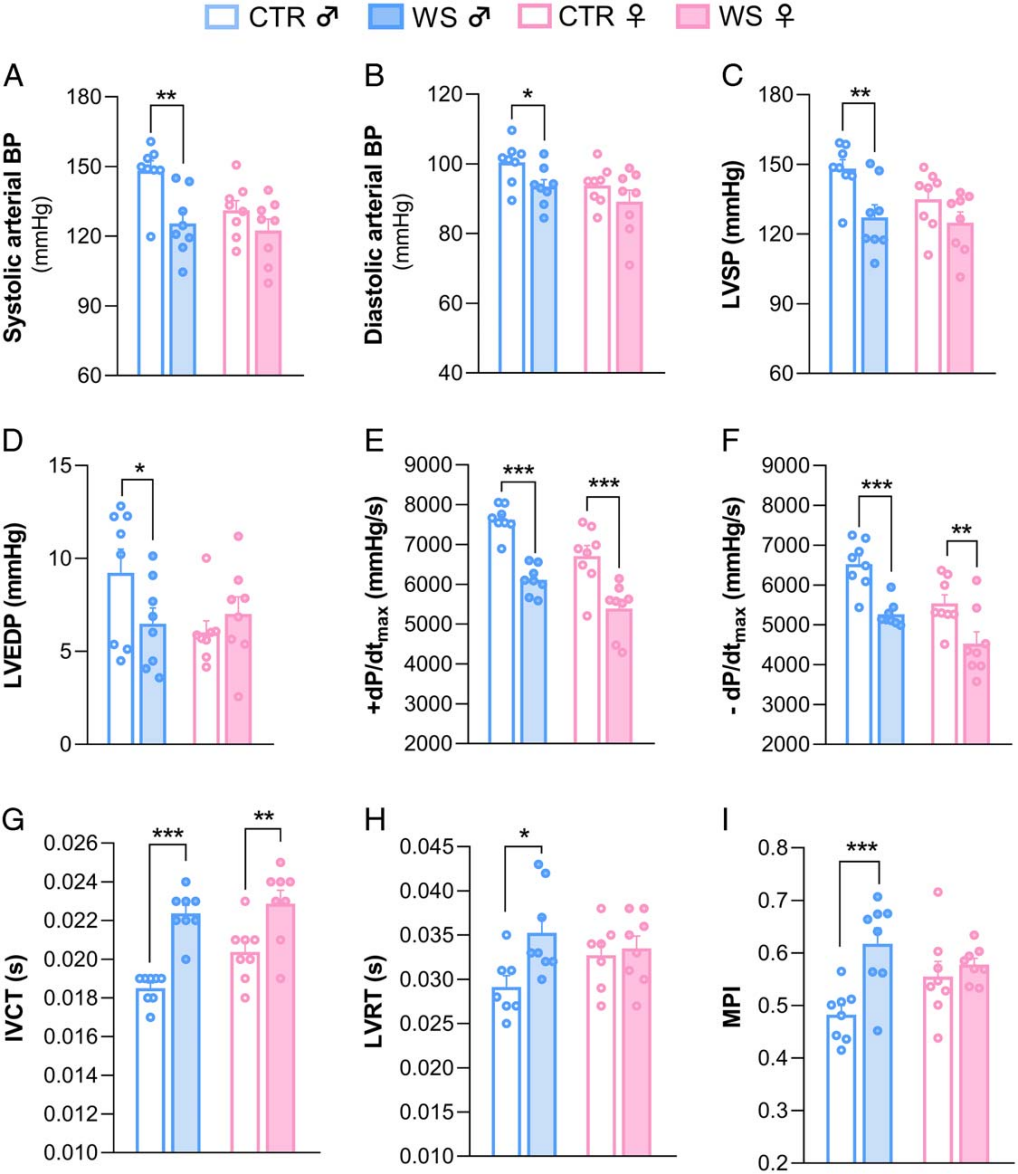
Hemodynamic parameters. (A) Systolic arterial blood pressure (BP), (B) diastolic arterial BP, (C) left ventricular systolic pressure (LVSP), (D) left ventricular end-diastolic pressure (LVEDP), (E) maximal rate of ventricular pressure rise (+dP/dt_max_), (F) maximal rate of ventricular pressure decline (−dP/dt_max_), (G) isovolumic contraction time (IVCT), (H) left ventricular relaxation time (LVRT), and (I) myocardial performance index (MPI) in rats of both sexes (♂: males; ♀: females) exposed to repeated episodes of witness stress (WS) or control procedure (CTR) (*n*=8 per group). Data are reported as means±SEM. Only within-sex effects are reported (*p<.05; **p<.01; ***p<.001). Color image is available only in online version.

Hemodynamic parameters are shown in Figure 2, while main effects of 2-way ANOVAs are reported in Table S3, Supplemental Digital Content, http://links.lww.com/PSYMED/B99.

WS male rats had a significantly lower arterial systolic (*p*=.002, Figure 2A) and diastolic (*p*=.050, Figure 2B) blood pressure compared with their same-sex CTRs, while no differences were found between WS and CTR females. Similarly, WS male rats displayed a significantly lower LVSP (*p*=.003, Figure 2C) and LVEDP (*p*=.049, Figure 2D) compared with CTR males, while no significant differences were detected between WS and CTR females. Importantly, WS exposure significantly affected several parameters of contractile function in rats of both sexes compared with their same-sex CTRs, but the magnitude of such effects (reported as Cohen's *d*) was greater in males in all instances. Specifically, WS rats showed significantly (*P*<0.010) lower maximal rates of left ventricular pressure rise (+dP/dt_max_, Figure 2E; Cohen's *d* females, *d*=1.99 vs. Cohen's *d* males, *d*= 4.42) and decline (−dP/dt_max_, Figure 2F, Cohen's *d* females, *d*=1.45 vs. Cohen's *d* males, *d*= 2.82) during isovolumic contraction, and a significant (*p*<.001) prolongation of IVCT (Figure 2G; Cohen's *d* females, *d*=1.53 vs. Cohen's *d* males, *d*=4.24) compared with their respective CTRs. Male WS rats also showed a significantly longer LVRT (Figure 2H) compared with male CTRs (*p*=.038), while no significant differences were found between WS and CTR females. No significant differences between groups and sexes were found for ejection time (male CTR: 0.1±0.002 s; male WS: 0.09±0.003 s; female CTR: 0.1±0.002 s; female WS: 0.1±0.001 s). Lastly, the MPI was calculated as an index of global contractile performance, where higher values of the ratio mean worse performance. As shown in Figure 2I, the MPI ratio was significantly higher in WS males compared with CTR males (+28%, *p*<.001), whereas no significant changes were found between WS and CTR females.

### Sex-specific Effects of WS on miRNA Cardiac Levels

**FIGURE 3:**
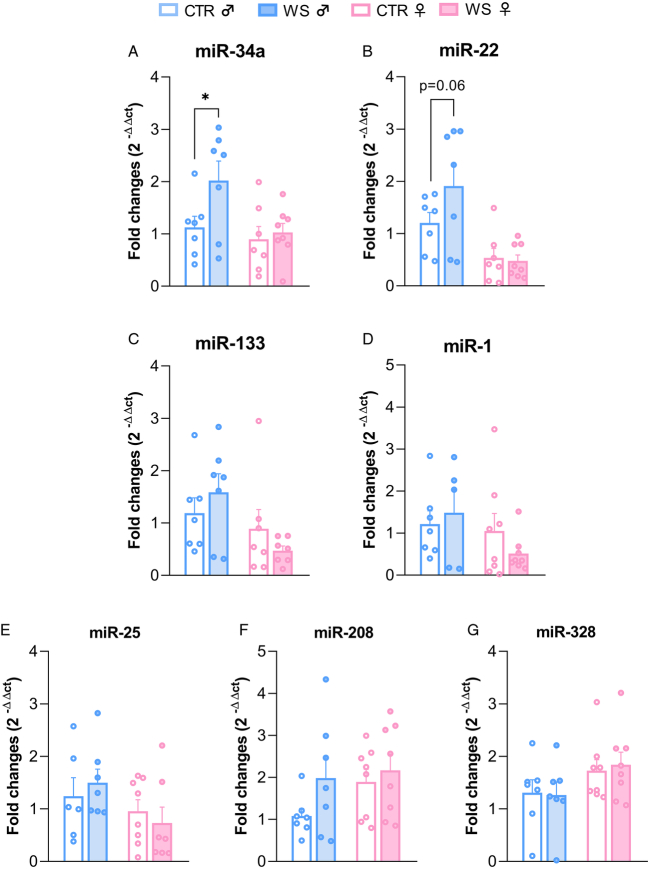
Cardiac miRNA (miR) levels. Relative expression (fold changes) of miR-34a (A), miR-22 (B), miR-133 (C), miR-1 (D), miR-25 (E), miR-208 (F), miR-328, (G) in the hearts of rats of both sexes (♂: males; ♀: females; exposed to repeated episodes of witness stress (WS) or control procedure (CTR) (*n* = 8 per group). The male CTR group served as a reference group for the other groups. Data are reported as means±SEM. Only within-sex effects are reported (*p<.05 ). Color image is available only in online version.

Figure 3 represents relative cardiac expression levels of candidate miRNAs that have been previously implicated in cardiac electromechanical dysfunction. ANOVA yielded a significant effect of “group” (*F*_(1,25)_=3.96, *p*=.050) and “sex” (*F*_(1,25)_=5.59, *p*=.026) for cardiac miR-34a expression level (Figure 3A). Specifically, WS male rats showed significantly higher miR34a expression compared with both male CTRs (*p*=.011) and WS female rats (*p*=.023). Two-way ANOVAs indicated a significant effect of “sex” for miR-22 (*F*_(1,25)_=17.31, *p*<.001) and miR-133 (*F*_(1,25)_=5.63, *p*=.026), with WS male rats displaying higher levels of these 2 miRNAs compared with WS female rats (miR-22: *p*<.001, Figure 3B; miR-133: *p*=.014, Figure 3C). The relative expression of miR-22 in WS male rats was also higher than in male CTRs, although this difference did not reach full statistical significance (*p*=.06, Figure 3B). No significant group or sex differences were found for miR-1, miR-25, miR-208, and miR-328.

### Sex-specific Effects of WS on Sirtuin-1 and Connexin-43 Cardiac Levels

**FIGURE 4:**
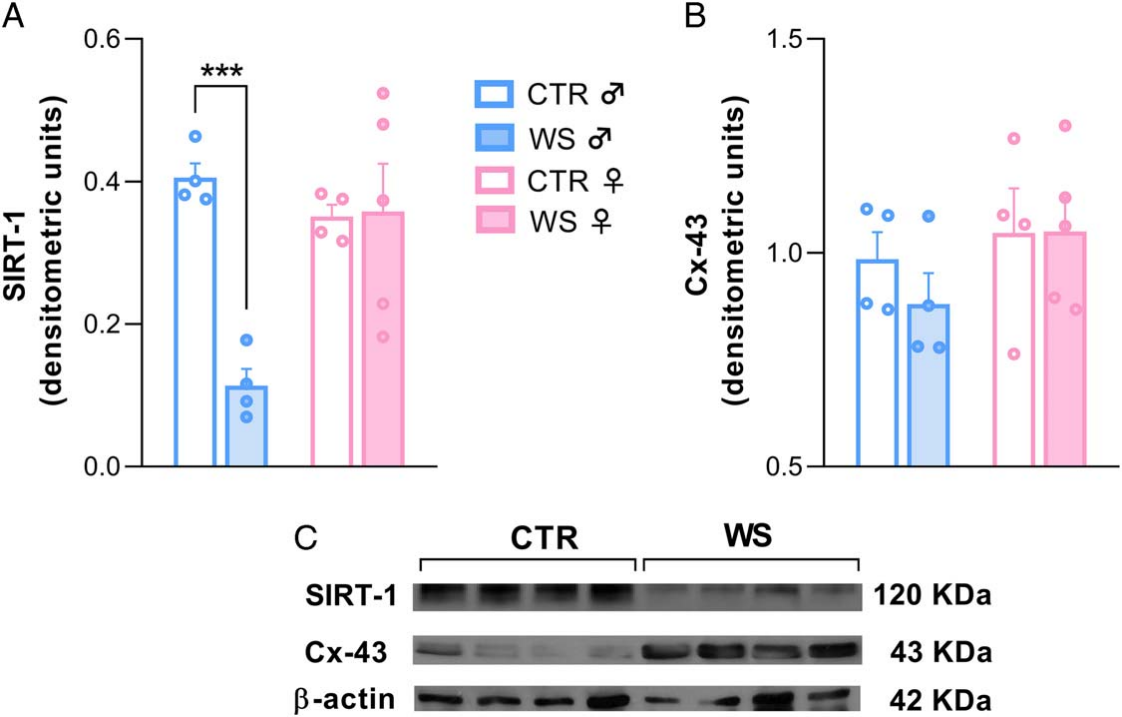
Cardiac sirtuin-1 (SIRT-1) and connexin-43 (Cx-43) levels. Protein expression levels (expressed as densitometric units) of SIRT-1 (A) and Cx-43 (B) in the heart tissue of rats of both sexes (♂: males; ♀: females) exposed to repeated episodes of witness stress (WS) or control procedure (CTR) (*n*=4/5 per group). Densitometric analyses, normalized to β-actin, measured protein expression levels. (C) Example of electrophoretic separation and immunodetection bands of SIRT-1, Cx-43 and β-actin in male rats. Data are reported as means±SEM. ***p<.001. Color image is available only in online version.

Western blot analyses revealed significantly lower SIRT-1 levels in WS males compared with their same-sex CTRs (*t*=9.466, *p*<.001), while no significant differences were found among females (Figure 4A). In contrast, Cx-43 protein content did not differ between WS and CTR groups in both sexes (Figure 4B). No significant differences in β-actin levels were found between groups (Figure S1, Supplemental Digital Content, http://links.lww.com/PSYMED/B99).

## DISCUSSION

In this study, we demonstrated the emergence of sex-specific adverse effects of repeated WS exposure on cardiac electrical and contractile function, which were associated with sex-specific cardiac molecular changes in adult rats. Specifically, male rats, but not females, showed an increased vulnerability to pharmacologically induced arrhythmias after repeated WS. Also, signs of cardiac contractile impairment were found in both sexes after WS, but to a greater extent in male rats. Notably, our results suggest a role for miR-34a and miR-22 in the epigenetic control of SIRT-1 in the male rat heart upon WS exposure, which may represent a pathophysiological mechanism underlying greater electromechanical vulnerability in stressed males.

In a recent study, we demonstrated that repeated WS exposure induces behavioral and neuroendocrine (ie, corticosterone) stress responses in wild-type Groningen rats of both sexes.^26^ Here, male rats exposed to repeated WS exhibited a significantly higher arrhythmic burden following β-adrenergic stimulation with isoproterenol compared with their female counterparts. Interestingly, WS did not affect the electrical stability of the hearts of female rats. The increased arrhythmic vulnerability found in male rats was primarily ascribable to a significantly larger incidence of premature atrial beats and atrioventricular blocks, which typically derive from impairments in the conduction of the electrical impulse. Intriguingly, atrioventricular blocks have been demonstrated to be spontaneously more frequent in male rats than females,^52^ suggesting inherent sex differences in the cardiac conduction system. For example, females generally exhibit greater sinoatrial node automaticity and enhanced atrioventricular node function,^53,54^ which may confer protection against conduction abnormalities under stress conditions. Furthermore, social stress could induce electrophysiological changes that may favor arrhythmogenesis in a sex-specific manner: for example, reduced myocardial refractoriness and decreased conduction velocity, which are major determinants of arrhythmogenesis, were previously found in male rats exposed to repeated episodes of social defeat.^13^ A larger expression of connexins, which form gap-junctions to facilitate the propagation of electrical activity through cardiomyocytes, may explain the resistance of the female heart to arrhythmias. Specifically, in a previous study, male rats have been reported to exhibit lower levels of connexin-43 under both basal and pathological conditions compared with females.^55^ However, in this study, we did not detect any sex difference in the expression of connexin-43, both in CTR and WS rats, suggesting that other mechanisms may be involved.

In addition to electrical abnormalities, WS exposure was also associated with alterations of several hemodynamic parameters, indicating an impairment of cardiac contractile performance in both sexes, but to a greater extent in males. Indeed, male WS rats exhibited lower systolic and diastolic pressures at both aortic and ventricular levels compared with their respective CTRs, whereas no significant effects of WS were found in females. Moreover, we detected a significantly longer left-ventricular relaxation time only in male witnesses, which were also characterized by a higher myocardial performance index, indicating a worsening of the global myocardial contractile performance.^46^ Although repeated episodes of WS impacted the contractile properties of the female heart, as evidenced by a significantly lower maximal rate of left ventricular pressure rise and decline during isovolumic contraction and an increased isovolumetric contraction time compared with controls, these alterations appeared to be milder than those observed in males. However, it should be noted that female CTR rats exhibited lower arterial and left ventricular pressures than their male counterparts, in line with the well-documented sex differences in the control of blood pressure in both rodents and humans.^56–58^ At the same time, we cannot rule out the possibility that female animals were more sensitive to the control condition adopted in this study (ie, empty cage exposure), therefore masking potential effects of WS on arterial and left ventricular pressures. Nevertheless, the results of the hemodynamic analyses suggest that repeated WS exposure results in a more pronounced decline of the mechanical performance of the male heart.

The second objective of the current study was to explore whether cardiac electrical and contractile impairments after WS were associated with changes in the cardiac expression of candidate miRNAs previously implicated in cardiac (patho)physiology: mir-1, mir-22, miR-25, miR34a, miR-133, miR-208, and miR-328.^12,34–38^ Our results revealed that the adverse remodeling of the male heart after WS was associated with a larger cardiac expression of miR-22 and miR-34a. Conversely, no changes were found in the hearts of female rats exposed to WS. miR-22, which is highly expressed in the heart, has been suggested to play a key role in cardiac remodeling after stress exposure, particularly in contractile dysfunction and hypertrophy.^36,59^ miR-34a has already been implicated in both cardiac pathology and psychosocial stress responses, representing a putative link between stress and CVD risk. In our previous work, we reported an association between psychosocial stress and elevated cardiac miR-34a levels in male rats exposed to chronic social defeat,^12^ but no changes in miR-34a levels in female rats subjected to repeated WS exposure.^33^ The current study in rats exposed to the same social stressor (WS) reinforces this sex-specific pattern, as miR-34a levels were significantly elevated only in males. Intriguingly, miR-34a and miR-22 share a common molecular target: SIRT1. This protein, through its NAD^+^-dependent deacetylase activity, regulates a wide range of cellular processes and is involved in multiple signaling pathways.^60^ Growing evidence suggests that SIRT-1 plays a crucial role in cardiovascular physiology. While primarily targeting histones, SIRT-1 also acts on other proteins, including p53, NF-κB, FoxO, and eNOS.^61^ Through these interactions, SIRT-1 exerts many cardiovascular functions, including mitigation of inflammation and oxidative stress, inhibition of cardiomyocytes apoptosis and myocardial energy balance maintenance,^62^ playing an essential protective activity in the heart. Therefore, given the increased cardiac expression of miR-22 and miR-34a in male WS rats, we investigated whether cardiac levels of their common molecular target SIRT1 were reduced. Confirming our hypothesis, male WS rats exhibited decreased cardiac SIRT1 levels compared with their CTRs, while WS females did not show any significant alterations. These results suggest that increased expression of miR-22 and miR-34a may play a role in the epigenetic regulation of SIRT1 expression in male rats with a history of WS. This may represent a potential mechanism by which repeated WS exposure induces greater adverse electromechanical remodeling in male rats. However, further studies are needed to confirm the causal relationship between reduced SIRT1 expression and cardiac vulnerability to stress in male rats. Lastly, a higher expression of cardiac miR-133, which has been linked to electrical remodeling in heart failure,^34^ was found in male WS rats compared with WS females, but without a significant effect of stress exposure in either sex.

## CONCLUSIONS

Despite the growing interest in exploring sex differences across various fields of biomedical research, the extent to which social stress impacts cardiac function in a sex-specific manner remains unclear. The results of the current study offer valuable translational insights into sex differences in the negative cardiac consequences of social stress exposure and the underlying molecular underpinnings. Male adult rats demonstrated a higher susceptibility to cardiac electrical and mechanical impairments following repeated WS compared with age-matched females. To explain these sex differences, 2 main hypotheses can be put forward. First, sex hormones may have played a protective role against the development of adverse cardiac remodeling in adult female rats, as reported in humans.^63^ For example, epidemiological studies have demonstrated that the incidence and severity of CVD increase in postmenopausal women, coinciding with declines in ovarian progesterone and estrogens.^64^ However, it must be noted that a previous study in female rats demonstrated that while behavioral and cardiovascular responses to WS are independent from the specific estrous cycle stage, the presence of ovarian hormones is essential for behavioral, inflammatory and cardiovascular susceptibility to WS.^25^ In our study, the estrous cycle phase was not assessed, which may be seen as a limitation. Another hypothesis involves protective mechanisms related to sex differences in cardiac vagal tone. In fact, previous studies in the same rat strain of the current investigation have shown that (i) resting measures of heart rate variability, a surrogate measure of cardiac vagal modulation, predict vulnerability to cardiac arrhythmias in males, and that (ii) female rats show higher heart rate variability than males, which replicates human findings.^43,65^ Future studies also in other rat strains (eg, Wistar or Sprague Dawley rats) are required to confirm our findings and determine the influence of ovarian hormones and/or heart rate variability on individual and sex differences in WS-induced cardiac remodeling.

Importantly, the present findings suggest specific molecular pathways, involving miRNA-mediated control of SIRT-1, which may drive electromechanical changes in the heart of socially stressed male rats. Therefore, therapeutic approaches aimed at modulating specific miRNAs (ie, miR-34a/miR-22) and/or restoring SIRT-1 expression could mitigate stress-induced cardiac damage particularly in males. Future investigations should continue to unravel sex-specific molecular pathways underlying cardiovascular abnormalities after psychosocial stress exposure. This knowledge will refine sex-specific preventive and therapeutic strategies, ultimately improving stress-related cardiovascular health outcomes for both sexes.

## Supplementary Material

## References

[1] Helman TJ Headrick JP Stapelberg NJC Braidy N. The sex-dependent response to psychosocial stress and ischaemic heart disease. Front Cardiovasc Med. 2023;10:1072042. 37153459 10.3389/fcvm.2023.1072042 PMC10160413

[2] Medina de Chazal H Del Buono MG Keyser-Marcus L Ma L Moeller FG Berrocal D. Stress cardiomyopathy diagnosis and treatment. J Am Coll Cardiol. 2018;72:1955–1971. 30309474 10.1016/j.jacc.2018.07.072 PMC7058348

[3] Vaccarino V Shah AJ Rooks C Ibeanu I Nye JA Pimple P. Sex differences in mental stress–induced myocardial ischemia in young survivors of an acute myocardial infarction. Psychosom Med. 2014;76:171–180. 24608039 10.1097/PSY.0000000000000045 PMC4008686

[4] Vaccarino V Wilmot K Mheid I Al Ramadan R Pimple P Shah AJ. Sex differences in mental stress-induced myocardial ischemia in patients with coronary heart disease. J Am Heart Assoc. 2016;5:e003630. 27559072 10.1161/JAHA.116.003630 PMC5079026

[5] Chumaeva N Hintsanen M Juonala M Raitakari OT Keltikangas-Järvinen L. Sex differences in the combined effect of chronic stress with impaired vascular endothelium functioning and the development of early atherosclerosis: the Cardiovascular Risk in Young Finns study. BMC Cardiovasc Disord. 2010;10:34. 20624297 10.1186/1471-2261-10-34 PMC2912787

[6] Weidner G. Why do men get more heart disease than women? An international perspective. J Am Coll Health. 2000;48:291–294. 10863872 10.1080/07448480009596270

[7] Sgoifo A Carnevali L Grippo AJ. The socially stressed heart. Insights from studies in rodents. Neurosci Biobehav Rev. 2014;39:51–60. 24373860 10.1016/j.neubiorev.2013.12.005

[8] Barbetti M Vilella R Dallabona C Gerra MC Bocchi L Ielpo D. Decline of cardiomyocyte contractile performance and bioenergetic function in socially stressed male rats. Heliyon. 2022;8:e11466. 36387533 10.1016/j.heliyon.2022.e11466 PMC9660606

[9] Gao X Kim S Zhao T Ren M Chae J. Social defeat stress induces myocardial injury by modulating inflammatory factors. J Int Med Res. 2020;48:300060520936903. 32687424 10.1177/0300060520936903 PMC7372629

[10] Wideman CH Cierniak KH Sweet WE Moravec CS Murphy HM. An animal model of stress-induced cardiomyopathy utilizing the social defeat paradigm. Physiol Behav. 2013;120:220–227. 23962681 10.1016/j.physbeh.2013.08.017

[11] Yoshida Y Yajima Y Kawakami K Nakamura S Tsukahara T Oishi K. Salivary microRNA and metabolic profiles in a mouse model of subchronic and mild social defeat stress. Int J Mol Sci. 2022;23:14479. 36430957 10.3390/ijms232214479 PMC9692636

[12] Andolina D Savi M Ielpo D Barbetti M Bocchi L Stilli D. Elevated miR-34a expression and altered transcriptional profile are associated with adverse electromechanical remodeling in the heart of male rats exposed to social stress. Stress. 2021;24:621–634. 34227918 10.1080/10253890.2021.1942830

[13] Carnevali L Trombini M Rossi S Graiani G Manghi M Koolhaas JM. Structural and electrical myocardial remodeling in a rodent model of depression. Psychosom Med. 2013;75:42–51. 23257930 10.1097/PSY.0b013e318276cb0d

[14] Arias-de la Torre J Vilagut G Ronaldson A Serrano-Blanco A Martín V Peters M. Prevalence and variability of current depressive disorder in 27 European countries: a population-based study. Lancet Public Health. 2021;6:e729–e738. 33961802 10.1016/S2468-2667(21)00047-5 PMC8460452

[15] Zhao J Ye L Liu Z Cui Y Deng D Bai S. Protective effects of resveratrol on adolescent social isolation-induced anxiety-like behaviors via modulating nucleus accumbens spine plasticity and mitochondrial function in female rats. Nutrients. 2022;14:4542. 36364807 10.3390/nu14214542 PMC9656193

[16] Kumari A Singh P Baghel MS Thakur MK. Social isolation mediated anxiety like behavior is associated with enhanced expression and regulation of BDNF in the female mouse brain. Physiol Behav. 2016;158:34–42. 26921097 10.1016/j.physbeh.2016.02.032

[17] Keesom SM Morningstar MD Sandlain R Wise BM Hurley LM. Social isolation reduces serotonergic fiber density in the inferior colliculus of female, but not male, mice. Brain Res. 2018;1694:94–103. 29763575 10.1016/j.brainres.2018.05.010

[18] Nowacka MM Paul-Samojedny M Bielecka AM Obuchowicz E. Chronic social instability stress enhances vulnerability of BDNF response to LPS in the limbic structures of female rats: a protective role of antidepressants. Neurosci Res. 2014;88:74–83. 25173454 10.1016/j.neures.2014.08.008

[19] Baranyi J Bakos N Haller J. Social instability in female rats: the relationship between stress-related and anxiety-like consequences. Physiol Behav. 2005;84:511–518. 15811385 10.1016/j.physbeh.2005.01.005

[20] Harris AZ Atsak P Bretton ZH Holt ES Alam R Morton MP. A novel method for chronic social defeat stress in female mice. Neuropsychopharmacology. 2017;43:1276–1283. 29090682 10.1038/npp.2017.259 PMC5916350

[21] Takahashi A Chung JR Zhang S Zhang H Grossman Y Aleyasin H. Establishment of a repeated social defeat stress model in female mice. Sci Rep. 2017;7:12838. 28993631 10.1038/s41598-017-12811-8 PMC5634448

[22] Patki G Solanki N Salim S. Witnessing traumatic events causes severe behavioral impairments in rats. Int J Neuropsychopharmacol. 2014;17:2017–2029. 24887568 10.1017/S1461145714000923 PMC4318493

[23] Warren BL Vialou VF Iñiguez SD Alcantara LF Wright KN Feng J. Neurobiological sequelae of witnessing stressful events in adult mice. Biol Psychiatry. 2013;73:7–14. 22795644 10.1016/j.biopsych.2012.06.006 PMC3498570

[24] Finnell JE Lombard CM Padi AR Moffitt CM Wilson LB Wood CS. Physical versus psychological social stress in male rats reveals distinct cardiovascular, inflammatory and behavioral consequences. PLoS One. 2017;12:e0172868. 28241050 10.1371/journal.pone.0172868 PMC5328366

[25] Finnell JE Muniz BL Padi AR Lombard CM Moffitt CM Wood CS. Essential role of ovarian hormones in susceptibility to the consequences of witnessing social defeat in female rats. Biol Psychiatry. 2018;84:372–382. 29544773 10.1016/j.biopsych.2018.01.013 PMC6067999

[26] Barbetti M Sgoifo A Carnevali L. Sex-specific behavioral, cardiac, and neuroendocrine responses to repeated witness social stress in adult rats. Physiol Behav. 2024;287:114702. 39332593 10.1016/j.physbeh.2024.114702

[27] Du J Li M Huang Q Liu W Li W qun Li Y jian. The critical role of microRNAs in stress response: therapeutic prospect and limitation. Pharmacol Res. 2019;142:294–302. 30553824 10.1016/j.phrs.2018.12.007

[28] Sessa F Salerno M Esposito M Cocimano G Pomara C. miRNA dysregulation in cardiovascular diseases: current opinion and future perspectives. Int J Mol Sci. 2023;24:5192. 36982265 10.3390/ijms24065192 PMC10048938

[29] Chattopadhyay A Tak H Anirudh J Naick BH. Meta-analysis of circulatory mitomiRs in stress response: unveiling the significance of miR-34a and miR-146a. Gene. 2024;912:148370. 38490506 10.1016/j.gene.2024.148370

[30] Lo Iacono L Ielpo D Accoto A Di Segni M Babicola L D’Addario SL. MicroRNA-34a regulates the depression-like behavior in mice by modulating the expression of target genes in the dorsal Raphè. Mol Neurobiol. 2020;57:823–836. 31482401 10.1007/s12035-019-01750-2

[31] Haramati S Navon I Issler O Ezra-Nevo G Gil S Zwang R. microRNA as repressors of stress-induced anxiety: the case of amygdalar miR-34. J Neurosci. 2011;31:14191–14203. 21976504 10.1523/JNEUROSCI.1673-11.2011 PMC6623664

[32] Andolina D Di Segni M Accoto A Lo Iacono L Borreca A Ielpo D. MicroRNA-34 contributes to the stress-related behavior and affects 5-HT prefrontal/GABA amygdalar system through regulation of corticotropin-releasing factor receptor 1. Mol Neurobiol. 2018;55:7401–7412. 29417477 10.1007/s12035-018-0925-z

[33] Barbetti M Vilella R Naponelli V Bilotti I Magistrati M Dallabona C. Repeated witness social stress causes cardiomyocyte contractile impairment and intracellular Ca2+ derangement in female rats. Physiol Behav. 2023;271:114339. 37625474 10.1016/j.physbeh.2023.114339

[34] Belevych AE Sansom SE Terentyeva R Ho HT Nishijima Y Martin MM. MicroRNA-1 and -133 increase arrhythmogenesis in heart failure by dissociating phosphatase activity from RyR2 complex. PLoS One. 2011;6:e28324. 22163007 10.1371/journal.pone.0028324 PMC3232211

[35] Wahlquist C Jeong D Rojas-Muñoz A Kho C Lee A Mitsuyama S. Inhibition of miR-25 improves cardiac contractility in the failing heart. Nature. 2014;508:531–535. 24670661 10.1038/nature13073 PMC4131725

[36] Gurha P Abreu-Goodger C Wang T Ramirez MO Drumond AL Van Dongen S. Targeted deletion of MicroRNA-22 promotes stress-induced cardiac dilation and contractile dysfunction. Circulation. 2012;125:2751–2761. 22570371 10.1161/CIRCULATIONAHA.111.044354 PMC3503489

[37] Huang XH Li JL Li XY Wang SX Jiao ZH Li SQ. miR-208a in cardiac hypertrophy and remodeling. Front Cardiovasc Med. 2021;8:773314. 34957257 10.3389/fcvm.2021.773314 PMC8695683

[38] Lu Y Zhang Y Wang N Pan Z Gao X Zhang F. MicroRNA-328 contributes to adverse electrical remodeling in atrial fibrillation. Circulation. 2010;122:2378–2387. 21098446 10.1161/CIRCULATIONAHA.110.958967

[39] Sgoifo A Costoli T Meerlo P Buwalda B Pico-Alfonso MA De Boer S. Individual differences in cardiovascular response to social challenge. Neurosci Biobehav Rev. 2005;29:59–66. 15652255 10.1016/j.neubiorev.2004.07.001

[40] de Jong JG van der Vegt BJ Buwalda B Koolhaas JM. Social environment determines the long-term effects of social defeat. Physiol Behav. 2005;84:87–95. 15642611 10.1016/j.physbeh.2004.10.013

[41] Percie du Sert N Hurst V Ahluwalia A Alam S Avey MT Baker M. The ARRIVE guidelines 2.0: updated guidelines for reporting animal research. BMJ Open Science. 2020;44:242 10.1136/bmjos-2020-100115 PMC7610906 34095516

[42] Sgoifo A Stilli D Medici D Gallo P Aimi B Musso E. Electrode positioning for reliable telemetry ECG recordings during social stress in unrestrained rats. Physiol Behav. 1996;60:1397–1401. 8946481 10.1016/s0031-9384(96)00228-4

[43] Carnevali L Statello R Sgoifo A. Resting heart rate variability predicts vulnerability to pharmacologically-induced ventricular arrhythmias in male rats. J Clin Med. 2019;8:655. 31083474 10.3390/jcm8050655 PMC6572182

[44] Curtis MJ Hancox JC Farkas A Wainwright CL Stables CL Saint DA. The Lambeth Conventions (II): guidelines for the study of animal and human ventricular and supraventricular arrhythmias. Pharmacol Ther. 2013;139:213–248. 23588158 10.1016/j.pharmthera.2013.04.008

[45] Grippo AJ Moffitt JA Sgoifo A Jepson AJ Bates SL Chandler DL. The integration of depressive behaviors and cardiac dysfunction during an operational measure of depression. Psychosom Med. 2012;74:612–619. 22753634 10.1097/PSY.0b013e31825ca8e5 PMC3392416

[46] Salemi VMC Pires MD Cestari N Cestari IA Picard MH Leirner AA. Echocardiographic assessment of global ventricular function using the myocardial performance index in rats with hypertrophy. Artif Organs. 2004;28:332–337. 15084191 10.1111/j.1525-1594.2004.47350.x

[47] Schmittgen TD Livak KJ. Analyzing real-time PCR data by the comparative CT method. Nat Protoc. 2008;3:1101–1108. 18546601 10.1038/nprot.2008.73

[48] Boengler K Schulz R. Connexin 43 and mitochondria in cardiovascular health and disease. Adv Exp Med Biol. 2017;982:227–246. 28551790 10.1007/978-3-319-55330-6_12

[49] Matsushima S Sadoshima J. The role of sirtuins in cardiac disease. Am J Physiol Heart Circ Physiol. 2015;309:1375–1389. 10.1152/ajpheart.00053.2015 PMC4666968 26232232

[50] Yamakuchi M Ferlito M Lowenstein CJ. miR-34a repression of SIRT1 regulates apoptosis. Proc Natl Acad Sci USA. 2008;105:13421–13426. 18755897 10.1073/pnas.0801613105 PMC2533205

[51] Chen H Lu Q Fei X Shen L Jiang D Dai D. miR-22 inhibits the proliferation, motility, and invasion of human glioblastoma cells by directly targeting SIRT1. Tumor Biol. 2016;37:6761–6768. 10.1007/s13277-015-4575-8 26662303

[52] Pereira PJS Pugsley MK Troncy E Tan W Pouliot M Harper C. Incidence of spontaneous arrhythmias in freely moving healthy untreated Sprague-Dawley rats. J Pharmacol Toxicol Methods. 2019;99:106589. 31154034 10.1016/j.vascn.2019.106589

[53] Taneja T Windhagen Mahnert B Passman R Goldberger J Kadish A. Effects of sex and age on electrocardiographic and cardiac electrophysiological properties in adults. PACE. 2001;24:16–21. 11227963 10.1046/j.1460-9592.2001.00016.x

[54] Liu S Yuan S Kongstad O Olsson SB. Gender differences in the electrophysiological characteristics of atrioventricular conduction system and their clinical implications. Scand Cardiovasc J. 2001;35:313–317. 11771822 10.1080/140174301317116280

[55] Stauffer BL Sobus RD Sucharov CC. Sex differences in cardiomyocyte Connexin43 expression. J Cardiovasc Pharmacol. 2011;58:32–39. 21753256 10.1097/FJC.0b013e31821b70b4 PMC3136750

[56] Maris ME Melchert RB Joseph J Kennedy RH. Gender differences in blood pressure and heart rate in spontaneously hypertensive and Wistar-Kyoto rats. Clin Exp Pharmacol Physiol. 2005;32(1-2):35–39. 15730432 10.1111/j.1440-1681.2005.04156.x

[57] Onishi M Yamanaka K Miyamoto Y Waki H Gouraud S. Trpv4 involvement in the sex differences in blood pressure regulation in spontaneously hypertensive rats. Physiol Genomics. 2018;50:272–286. 29373075 10.1152/physiolgenomics.00096.2017

[58] Reckelhoff JF. Gender differences in the regulation of blood pressure. Hypertension. 2001;37:1199–1208. 11358929 10.1161/01.hyp.37.5.1199

[59] Huang ZP Wang DZ. miR-22 in cardiac remodeling and disease. Trends Cardiovasc Med. 2014;24:267–272. 25218673 10.1016/j.tcm.2014.07.005 PMC4171194

[60] Rahman S Islam R. Mammalian Sirt1: insights on its biological functions. Cell Commun Signal. 2011;9:11. 21549004 10.1186/1478-811X-9-11 PMC3103488

[61] Michan S Sinclair D. Sirtuins in mammals: insights into their biological function. Biochem J. 2007;404:1–13. 17447894 10.1042/BJ20070140 PMC2753453

[62] Ma L Li Y. SIRT1: role in cardiovascular biology. Clin Chim Acta. 2015;440:8–15. 25444742 10.1016/j.cca.2014.10.036

[63] Kan Y Peng YL Zhao ZH Dong ST Xu YX Ma XT. The impact of female sex hormones on cardiovascular disease: from mechanisms to hormone therapy. J Geriatr Cardiol. 2024;21:669–681. 38973823 10.26599/1671-5411.2024.06.003 PMC11224657

[64] Hayward C. The roles of gender, menopause and hormone replacement on cardiovascular function. Cardiovasc Res. 2000;46:28–49. 10727651 10.1016/s0008-6363(00)00005-5

[65] Carnevali L Barbetti M Statello R Williams DP Thayer JF Sgoifo A. Sex differences in heart rate and heart rate variability in rats: implications for translational research. Front Physiol. 2023;14:1170320. 37035663 10.3389/fphys.2023.1170320 PMC10080026

