# Supplementary material for: Sex Differences in the Adverse Electromechanical Remodeling of the Heart After Repeated Witness Stress in Adult Rats: Relationship With a Specific miRNA Signature
Source: Biopsychosoc Sci Med. 2025 May 23;88(1):37–47. doi: 10.1097/PSY.0000000000001406 (PMC12721695; doi:10.1097/PSY.0000000000001406)
Supplement: Supplementary file 1 [file psy-88-37-s001.docx]

**Supplemental Digital Content**

**Sex differences in the adverse electromechanical remodeling of the heart after repeated witness stress in adult rats: relationship with a specific miRNA signature**

Barbetti, Margherita PhD ^a^, Bilotti, Iolanda M.Sc. ^a^, Ielpo, Donald PhD ^b,c^, Frati, Caterina PhD ^a^, Naponelli, Valeria PhD ^d^, Andolina, Diego PhD ^b,c^, Lo Iacono, Luisa PhD ^b,e^, Sgoifo, Andrea PhD ^a^, Savi, Monia PhD ^a^, Carnevali, Luca PhD ^a^

^a^ Department of Chemistry, Life Sciences and Environmental Sustainability, University of Parma, Parma, Italy

^b^ Department of Psychology, Sapienza University, Rome, Italy

^c^ IRCCS Fondazione Santa Lucia, Rome, Italy

^d^ Department of Medicine and Surgery, University of Parma, Parma, Italy

^e^ Department of Translational Research and of New Surgical and Medical Technologies, University of Pisa, Pisa, Italy

**Table S1.** Total number of arrhythmic events for each category calculated during the hour that followed isoproterenol injection in male and female rats exposed to control (CTR: n=8/sex) or witness stress (WS: n=8/sex) procedures. Data are reported as means ± SEM.

| Arrhythmic categories | CTR males | WS males | CTR females | WS females |
| --- | --- | --- | --- | --- |
| Atrial premature beats | 2.5 ± 0.7 | 19.5 ± 3.3 *^ | 6.0 ± 1.0 | 5.9 ± 1.6 |
| Atrio-ventricular block | 0.3 ± 0.3 | 21.3 ± 6.2 *^ | 0.1 ± 0.1 | 0.6 ± 0.4 |
| Isolated premature ventricular complex | 35.4 ± 6.8 | 40.5 ± 11.9 | 53.0 ± 8.9 | 38.8 ± 6.6 |
| SALVO | 4.3 ± 2.1 | 3.4 ± 1.7 | 1.8 ± 0.5 | 1.5 ± 0.8 |
| Bigeminy | 0.6 ± 0.4 | 2.6 ± 1.9 | 0.5 ± 0.3 | 0.1 ± 0.1 |
| Trigeminy | 0.1 ± 0.1 | 1.3 ± 1.1 | 0.3 ± 0.2 | 0.0 ± 0.0 |
| Non-sustained ventricular tachycardia | 0.9 ± 0.5 | 0.6 ± 0.4 | 0.1 ± 0.1 | 0.0 ± 0.0 |
| Sustained ventricular tachycardia | 0.1 ± 0.1 | 0.3 ± 0.3 | 0.0 ± 0.0 | 0.0 ± 0.0 |
| Asystole | 0.1 ± 0.1 | 0.6 ± 0.4 | 0.3 ± 0.2 | 1.0 ± 0.7 |

Notes. * indicates a significant difference (p<0.05) with the respective CTR group; ^ indicates a significant difference (p<0.05) with the opposite sex.

**Table S2.** Heart rate values (HR) recorded the hour that preceded and the one that followed isoproterenol injection in male and female rats exposed to control (CTR, n=8/sex) or witness stress (WS, n=8/sex) procedures. Data are reported as means ± SEM.

| Heart Rate (bpm) | CTR males | WS males | CTR females | WS females |
| --- | --- | --- | --- | --- |
| Before injection | 383.44 ± 5.12 | 372.81 ± 6.62 | 407.52 ± 12.41 | 405.05 ± 11.16 ^ |
| After injection | 466.34 ± 9.60 * | 478.34 ± 8.19 * | 497.53 ± 4.94 * | 470.87 ± 14.11 * |

Notes. * indicates a significant difference (p<0.05) with before-injection values; ^ indicates a significant difference (p<0.05) with the opposite sex.

**Table S3.** Results of two-way ANOVAs with "group" (witness stress (WS) vs control (CTR)) and "sex" (males vs females) as between-subject factors hemodynamic parameters.

|  | Group | Sex | Group*Sex |
| --- | --- | --- | --- |
| Systolic arterial BP (mmHg) | F_(1,28)_=11.73, p=0.002 | F_(1,28)_=4.77, p=0.04 | F_(1,28)_=2.32, p=0.14 |
| Diastolic arterial BP (mmHg) | F_(1,28)_=5.86, p=0.02 | F_(1,28)_=5.17, p=0.03 | F_(1,28)_=0.22, p=0.64 |
| LVSP (mmHg) | F_(1,28)_=11.22, p=0.002 | F_(1,28)_=2.81, p=0.11 | F_(1,28)_=1.4, p=0.25 |
| LVEDP (mmHg) | F_(1,28)_=0.85, p=0.36 | F_(1,28)_=2.04, p=0.16 | F_(1,28)_=3.93, p=0.04 |
| +dP/dt_max_ (mmHg/s) | F_(1,28)_=50.89, p<0.001 | F_(1,28)_=17.07, p<0.001 | F_(1,28)_=0.26, p=0.61 |
| -dP/dt_max_ (mmHg/s) | F_(1,28)_=26.49, p<0.001 | F_(1,28)_=15.29, p<0.001 | F_(1,28)_=0.34, p=0.56 |
| IVCT (s) | F_(1,28)_=39.63, p<0.001 | F_(1,28)_=6.62, p=0.016 | F_(1,28)_=1.51, p=0.23 |
| LVRT (s) | F_(1,28)_=1.83, p=0.19 | F_(1,28)_=0.45, p=0.51 | F_(1,28)_=2.97, p=0.09 |
| Ejection time (s) | F_(1,28)_=2.73, p=0.11 | F_(1,28)_=0.003, p=0.96 | F_(1,28)_=1.21, p=0.28 |
| Time to +dP/dt_max_ (s) | F_(1,28)_=14.34, p<0.001 | F_(1,28)_=3.36, p=0.078 | F_(1,28)_=0.73, p=0.40 |
| MPI | F_(1,28)_=11.48, p=0.002 | F_(1,28)_=0.51, p=0.48 | F_(1,28)_=5.76, p=0.02 |

Notes. Abbreviations: BP: blood pressure; LVSP: left ventricular systolic pressure; LVEDP: left ventricular end-diastolic pressure; +dP/dt_max_: maximal rate of ventricular pressure rise; -dP/dt_max_: maximal rate of ventricular pressure decline; IVCT: isovolumic contraction time; LVRT: left ventricular relaxation time; MPI: myocardial performance index.

**
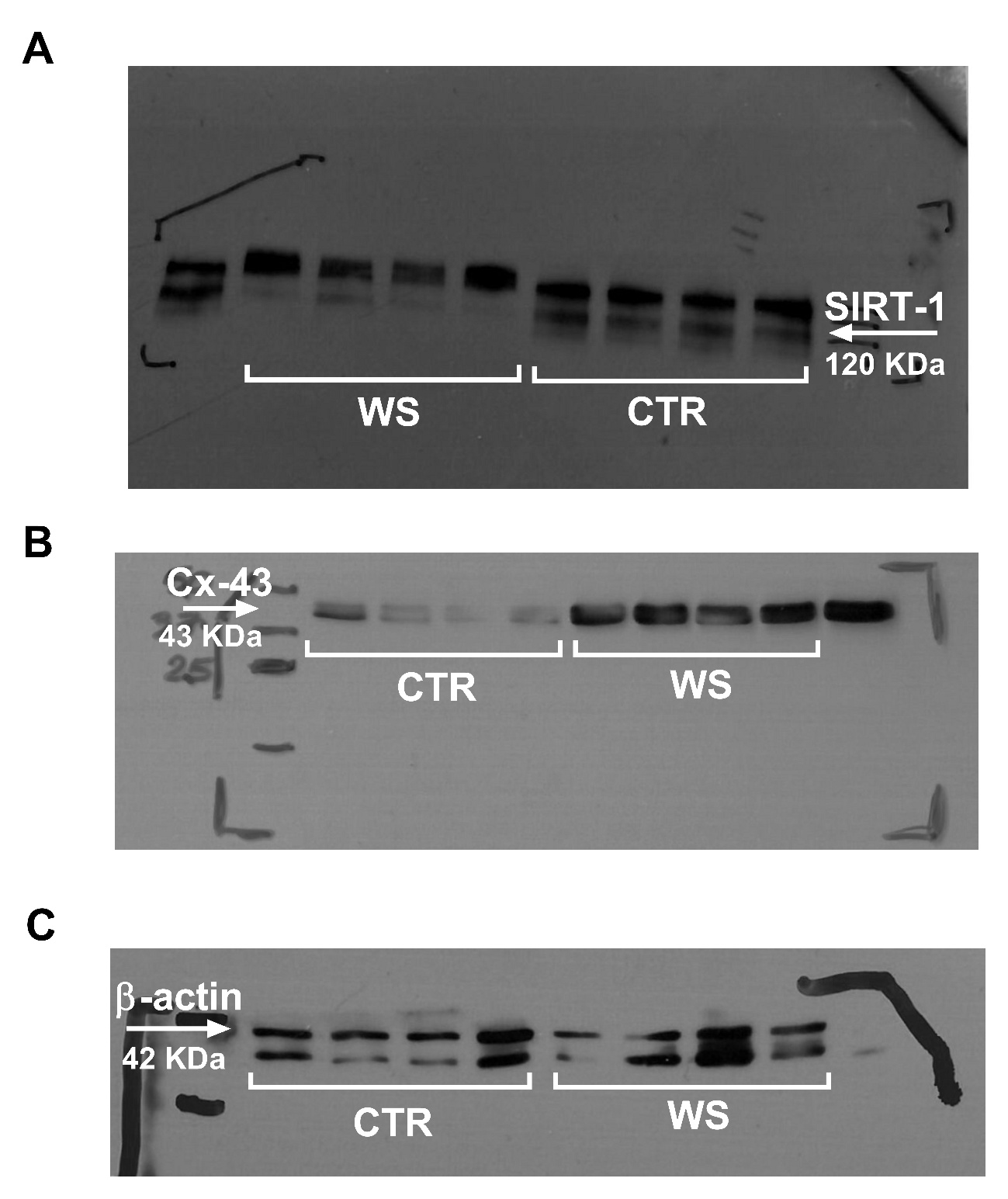
**

**Figure S1.** Electrophoretic separation and immunodetection bands of (A) SIRT-1, (B) Cx-43 and (C) β-actin.
